# Implementation of a double Gaussian source model for the BEAMnrc Monte Carlo code and its influence on small fields dose distributions

**DOI:** 10.1120/jacmp.v17i5.6372

**Published:** 2016-09-08

**Authors:** Edgardo Doerner, Paola Caprile

**Affiliations:** ^1^ Instituto de Física, Pontificia Universidad Católica de Chile Santiago Chile

**Keywords:** Monte Carlo, radiotherapy, BEAMnrc, small fields, source model

## Abstract

The shape of the radiation source of a linac has a direct impact on the delivered dose distributions, especially in the case of small radiation fields. Traditionally, a single Gaussian source model is used to describe the electron beam hitting the target, although different studies have shown that the shape of the electron source can be better described by a mixed distribution consisting of two Gaussian components. Therefore, this study presents the implementation of a double Gaussian source model into the BEAMnrc Monte Carlo code. The impact of the double Gaussian source model for a 6 MV beam is assessed through the comparison of different dosimetric parameters calculated using a single Gaussian source, previously commissioned, the new double Gaussian source model and measurements, performed with a diode detector in a water phantom. It was found that the new source can be easily implemented into the BEAMnrc code and that it improves the agreement between measurements and simulations for small radiation fields. The impact of the change in source shape becomes less important as the field size increases and for increasing distance of the collimators to the source, as expected. In particular, for radiation fields delivered using stereotactic collimators located at a distance of 59 cm from the source, it was found that the effect of the double Gaussian source on the calculated dose distributions is negligible, even for radiation fields smaller than 5 mm in diameter. Accurate determination of the shape of the radiation source allows us to improve the Monte Carlo modeling of the linac, especially for treatment modalities such as IMRT, were the radiation beams used could be very narrow, becoming more sensitive to the shape of the source.

PACS number(s): 87.53.Bn, 87.55.K, 87.56.B‐, 87.56.jf

## I. INTRODUCTION

Monte Carlo (MC) methods have been thoroughly used for different radiotherapy applications, especially for treatment planning and plan verification, proving to be one of the most accurate techniques to calculate dose distributions under clinical situations.^1^, ^2^ The latter is more evident when the dose has to be calculated in heterogeneous media and/or using superposition of small radiation fields, where other algorithms found in commercial treatment planning systems (TPS), such as pencil beam or collapsed cone convolution can fail to reproduce measured dose values.^3^, ^4^ However, the accuracy of a simulated dose distribution calculated using MC methods is very sensitive to the parameters that define the electron source.^(5)^ In particular, the spatial distribution of the source has an important influence on the shape of the penumbra and the dose distributions obtained for small radiation fields, due to source occlusion effects, among others.^6^, ^7^

Nowadays, there is a large number of general‐purpose MC platforms. Perhaps the most widely used in medical physics is EGSnrc.^3^, ^8^ An important special‐purpose code built on the EGSnrc platform is the user code BEAMnrc.^(9)^ This code is optimized to model the treatment head of radiotherapy linear accelerators (linacs) and includes a number of geometry and source subroutines, together with variance reduction techniques to enhance the efficiency of the simulation.^(10)^ In BEAMnrc the electron source of a linac is frequently modelled as a two‐dimensional Gaussian distribution.^11^, ^12^ The parameters for the distribution are traditionally determined through a commissioning process, in which a small set of measured and simulated distributions for several field sizes are compared iteratively until the parameters that minimize the difference are found.^(13)^

The shape of the photon source has been measured experimentally,^14^, ^15^ and its influence on the simulated dose distribution studied by several authors.^6^, ^16^ It has been found that the obtained photon dose distribution is very similar in shape to the electron source that originated it.^(17)^ Additionally, it has been suggested that the shape of the electron source can be better described by a mixed distribution consisting of two Gaussian components rather than a single Gaussian model, as assumed in previous studies.^(18)^

This work presents the implementation of a double Gaussian source model into the BEAMnrc code and its influence on the simulated dose distributions delivered by a 6 MV photon beam of a Siemens PRIMUS linac (Siemens Healthcare, Erlagen, Germany), with a special focus on small radiation fields. The parameters that define the electron beam distribution are derived from experimental measurements of the photon source. The influence of the shape of the source is evaluated by comparing different calculated dosimetric parameters/profiles, using the new source model, with MC calculations performed with a single Gaussian distribution, previously commissioned for this particular accelerator,^(13)^ and measurements.

## II. MATERIALS AND METHODS

### A. Double Gaussian source model

A symmetrical source model consisting on the sum of two Gaussian distributions was adopted and implemented into the BEAMnrc system. The distribution that defines the shape of the normalized electron source, as function of the radial position, can then be written as,

(1) $$f(r)=a_{1}e^{–r^{2}/2\sigma_{1}^{2}}+a_{2}e^{–r^{2}/2\sigma_{2}^{2}}$$

where $a_{1}$ and $a_{2}$ correspond to the weights of each Gaussian, with $a_{1}+a_{2}=1;\;\sigma_{1}$ and $\sigma_{2}$ define the width of each distribution. From this distribution, the starting positions of the incident electrons are obtained through random sampling. However, this function is not invertible. One approach to obtain a random sample $r^{*}$ is to use a probabilistic mixture model, where f(r) corresponds to a mixture distribution of a collection of two Gaussian components with associated probabilities (or weights) of occurrence, $P_{i}$. A member of this collection is then selected according to these weights and the desired sample is finally retrieved. Therefore, $r^{*}$ is obtained by performing the following steps.

1. First, the mixture weight of each Gaussian distribution is calculated from the area under the curve:
  (2) $$A_{i}=\int_{0}^{\infty }a_{i}e^{–r^{2}/2\sigma_{i}^{2}}\;r\;dr=a_{i}\sigma_{i}^{2}$$
  Therefore, the probability of occurrence $P_{i}$ corresponding to each mixture component equals to:
  (3) $$P_{i}=\frac{A_{i}}{A_{1}+A_{2}}$$
2. Then, one of the Gaussian distributions is selected using a random variable $R_{1}$, uniformly distributed over (0,1). If $R_{1}\leq P_{1}$, the first Gaussian distribution is chosen, otherwise the second distribution is taken.
3. As the selected Gaussian function is invertible, a random sample can be obtained using a “direct sampling” method.^(19)^ A second random variable $R_{2}$, uniformly distributed over (0,1), is used to calculate $r^{*}$ directly as

(4) $$r^{*}=\sqrt{–2\sigma_{i}^{2}\;ln(R_{2})}$$

The final result is a random radial position $r^{*}$ that follows the distribution f(r) from Eq. (1).

This model was introduced in the BEAMnrc user code as a source routine using a modified EGSnrc platform.^(20)^ The user must provide the $a_{i}$ and $\sigma_{i}$ parameters for each Gaussian distribution, in cm, indicating their weights and breadths, respectively. Alternatively to $\sigma_{i}$, the user can input the FWHM, also in cm, corresponding to each Gaussian. If the sum of the weights does not equal 1, the source routine automatically normalizes the distribution to the sum of the Gaussian weights. Additionally, the user can introduce the aforementioned parameters independently for X and Y directions in order to be able to model nonrotational symmetric electron sources.

In order to check the correct implementation of the source model, transverse electron fluence profiles were obtained. The profiles were generated by simulating a monoenergetic 6 MeV electron beam incident on a very thin air slab (0.001 cm thickness). A phase space file was scored just below the slab. This small slab thickness minimizes the probability of interaction of the incident electrons within the slab.^(11)^ The $a_{i}$ and $\sigma_{i}$ parameters for the double Gaussian model were obtained by fitting a double Gaussian distribution to an experimentally determined photon source for this linac,^(6)^ taking into consideration that the shape of the photon and electron sources are very similar.^(17)^

### B. Validation of the source model

In order to investigate the dosimetrical effects of the implementation of the new source model, a full MC simulation of a Siemens PRIMUS clinical linear accelerator (Siemens OCS, Concord, CA) for a 6 MV photon beam was performed using the BEAMnrc code. The simulations were carried out considering a single Gaussian source and the new double Gaussian source model. The results were compared then with experimental measurements.

The modeled accelerator includes the target, primary collimator, flattening filter, monitor chamber, collimating jaws, MLC, and, if needed, stereotactic cylindrical collimators. The variance reduction parameters in all the simulations were set according to Pena et al.^(13)^ An incident electron beam on the target with a Gaussian energy spectrum of 6 MeV mean and a FWHM of 14% was chosen for both beam sources as recommended by the manufacturer. For the new model, the parameters defining the source shape were set as described in the Material and Methods section A above. For the single Gaussian beam source, a FWHM of 0.10 cm was chosen following an automated commissioning procedure specifically designated to ensure best small field reproducibility.^(13)^ ECUT and PCUT values of 0.521 and 0.01 MeV, respectively, were used for all simulations. These parameters correspond to the electron and photon energy cutoffs that define the values at which the code stops tracking a particle. To improve the calculation efficiency, bremsstrahlung splitting with a photon split factor of 1000 was used. The splitting radius was defined at 100 cm of the source and its value was dependent on the field size. Phase space files were scored at 100 cm from the front face of the target. These phase space files were then applied to a homogeneous water phantom using the DOSXYZnrc user code in order to score output factors and dose profiles at a depth of 1.5 cm and SSD of 100 cm. For the BEAMnrc and DOSXYZnrc simulations $2\times 10^{7}$ and $2\times 10^{9}$ incident histories were used, respectively. The parameters used to validate our source model were the following: a) shape of the photon source, b) output factors, c) relative dose profiles, and d) penumbra extension.

### C. Shape of the photon source

The spatial distribution of the photon source of the linac has been experimentally determined using a procedure described by Caprile and Hartmann in 2009.^(6)^ It was found that the source had radial symmetry. The extended source was defined as the projection of all primary photons and head scatter contributions into a common plane, perpendicular to the beam axis and located at 100 cm from the isocenter. To determine the relative intensity distribution of the extended source, a slit‐method,^(14)^ based on the measurement of strip integrals of the source and the use of a CT image reconstruction technique, together with a fitting procedure for measured collimator factors, was used.

The BEAMnrc user code was used to calculate the distribution of the photon source, derived from our double Gaussian implementation and for the single Gaussian. This was done as follows. First, a phase space file was scored at 100 cm from the target with a field size of $10\times 10\;{\text{cm}}^{2}$. The position and direction of each particle scored in the phase space file was used to project them to the source plane. Then, an energy fluence versus position plot was obtained at this location and compared with the experimentally reconstructed source.

### D. Output factors

Output factors measurements were performed in a water tank (Model MP3, PTW Freiburg, Germany) using a diode detector (Type p, Model 6008, PTW Freiburg) with a sensitive volume of only $0.03\;{\text{mm}}^{3}$. The irradiation conditions were the following: detector located at 1.5 cm depth, with a SSD of 100 cm, for square fields with side lengths sizes ranging from 0.5 to 10 cm. The output factors were also calculated, using the DOSXYZnrc user code. The absorbed dose was scored at the point of interest, centered with respect to the beam axis at a depth of 1.5 cm depth in water with a voxel size of $0.1\times 0.1\times 0.1\;{\text{mm}}^{3}$. The absorbed dose was then corrected with respect to the dose scored at the monitor chamber of the PRIMUS accelerator, in order to take into account the collimator exchange effect in the accelerator head,^(21)^ and normalized with respect to the dose obtained for the $10\times 10\;{\text{cm}}^{2}$ reference field.

### E. Relative dose profiles

Lateral dose profiles were measured with the diode for various square and circular fields.

The latter were shaped by cylindrical collimators used for stereotactic radiosurgery, with aperture diameters ranging from 1 to 10 mm, positioned at 41 cm from the surface. The same experimental conditions indicated in the Materials and Methods section D were used to determine these dose profiles. Off‐axis profiles were calculated in a water phantom using the DOSXYZnrc user code with a row of voxels of $0.1\times 0.1\times 0.1\;{\text{mm}}^{3}$, centered with respect to the field axis and at a depth of 1.5 cm.

### F. Penumbra extension

The parameter used to quantify the steepness of the field penumbra was the distance between the 20% and 80% isodose lines. Calculated and measured results for the field penumbra were compared for a number of collimator settings for square fields, in the X and Y directions.

## III. RESULTS

### A. Spatial extension of the photon source

**Figure 1:**
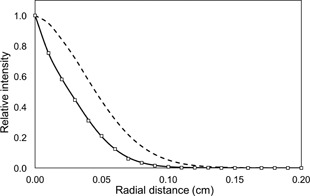
Reconstructions of the photon radiation source from an experimental procedure (empty squares) and MC calculations, with the double (solid line) and the single (dashed line) Gaussian sources.

Figure 1 shows reconstructed radial distributions of the photon radiation source. The spatial distribution of the photon source, derived from the double Gaussian source shows a FWHM of 0.051±0.002 cm that is in agreement with a value of 0.050±0.008 cm found experimentally, as expected. It is clear that the single Gaussian beam source strongly disagrees with the experimental results. For this last case, a FWHM of 0.100±0.003 cm was obtained. The uncertainty associated with the MC calculations was of ±0.5%. The agreement between the FWHM obtained for the photon source, derived from the double Gaussian electron source model and measurements is within 2%.

### B. Output factors

**Figure 2:**
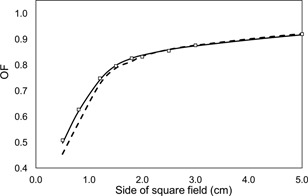
Comparison of measured (empty squares) and MC‐calculated OFs, with the double (solid line) and the single (dashed line) Gaussian sources.

Figure 2 shows a comparison between measured and calculated output factors for square fields, using the single and double Gaussian sources. All the curves obtained show a steep drop‐off for fields smaller than $3\times 3\;{\text{cm}}^{2}$. It can be seen that the results for the double Gaussian agree better with the measurements for the smaller fields. The uncertainty associated with the MC calculated output factors was of ±0.5%.

### C. Relative dose profiles

**Figure 3:**
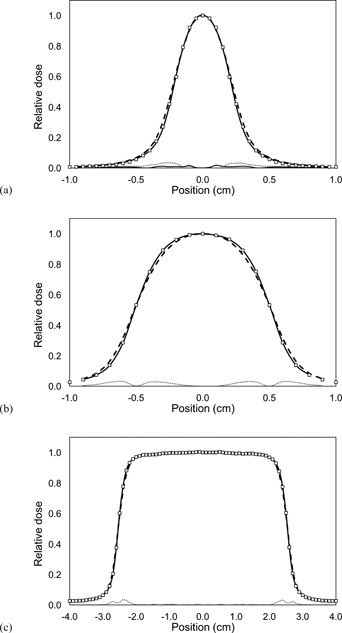
Measured (squares) and calculated (lines) relative dose profiles for square fields. MC calculations were carried out using a single Gaussian source with FWHM of 1.0 mm (dashed line) and the double Gaussian source (solid line): (a) $0.5\times 0.5\;{\text{cm}}^{2}$, (b) $1\times 1\;{\text{cm}}^{2}$, (c) $5\times 5\;{\text{cm}}^{2}$. Bottom lines represent the difference between measurements and calculations for the single (dotted line) and double (solid line) Gaussian sources.

3, 4 show the comparison of measured and calculated lateral dose profiles for different square and circular fields, respectively. For square fields the dose profiles obtained with the double Gaussian source show a better agreement with measurements, as shown in Fig. 3.

It was found that the maximum difference between measured and MC calculated dose profiles is of 1.7% for the double Gaussian source, whereas for the single Gaussian source a maximum difference of 3.3% was obtained. The difference between measured and calculated dose profiles decreases as the field size increases, an effect that can be observed in Fig. 3. It can be noted that this difference is negligible for the double Gaussian source, as shown in Fig. 3(b) and Fig. 3(c), where the difference line cannot be seen.

**Figure 4:**
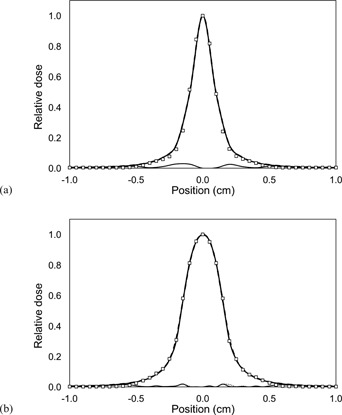
Measured (squares) and calculated (lines) relative dose profiles for circular fields. MC calculations were carried out using a single Gaussian source with FWHM of 1.0 mm (dashed line) and the double Gaussian source (solid line): (a) 1 mm, (b) 2 mm. Bottom lines represent the absolute difference between measurements and MC calculations using the single (dotted line) and the double (solid line) Gaussian sources.

For circular fields, it is not possible to distinguish the results obtained with the single and double Gaussian sources from the calculated dose profiles, even for the smallest collimator, as shown in Fig. 4, with the maximum difference between measured and MC calculated dose profiles below 3.0%. Nevertheless, MC calculated and measured dose profiles are in good agreement. The uncertainty associated with the MC calculations was of ±1.0%.

### D. Penumbra extension

Results for the field penumbra are listed in Table 1. The values, obtained for both calculations and measurements, present a steep drop‐off below the $5\times 5\;{\text{cm}}^{2}$ field. The values obtained for the penumbra are lower in the X direction, where the field sides are defined by the MLC. This behavior is due to the fact that the MLC is further from the source plane than the collimation jaws, which define the field sides in the Y direction. The uncertainty associated with MC simulations was 1.0%.

**Table 1:** Comparison of measured and MC calculated field penumbras p, in X and Y directions, for different square fields sizes (SF). MC calculations were carried out using the single and double Gaussian sources. For each calculation, the percentage difference from the measured value is indicated.

*SF (cm)*	*Axis*	*Meas. p (mm)*	*Single Gaussian*		*Double Gaussian*	
			*P (mm)*	$\delta_{\text{mean}}(\%)$	*P (mm)*	$\delta_{\text{mean}}(\%)$
0.5	X	1.9	2.0	8.3	1.8	−2.4
1.0	X	2.3	2.5	9.6	2.2	−2.5
5.0	X	2.7	2.9	8.6	2.6	−2.3
10.0	X	2.7	3.0	9.3	2.7	−2.1
0.5	Y	2.5	2.8	11.9	2.5	−2.3
1.0	Y	2.8	3.2	12.0	2.8	−2.6
5.0	Y	3.3	3.7	11.6	3.2	−2.8
10.0	Y	3.4	3.7	10.9	3.3	−2.2

## IV. DISCUSSION AND CONCLUSIONS

The implementation and validation of a double Gaussian source model into the BEAMnrc code and its influence on simulated dose distributions have been described. The selection of this specific source shape was based on the studies of Sterpin et al.^(17)^ and Chen et al.,^(18)^ which show that the shape of the electron source of a linac can be better described by a mixed distribution consisting of two Gaussian components, instead of the single Gaussian source model commonly used in Monte Carlo simulations. The increase in the execution time when the double Gaussian source model is used is negligible, being less than 0.5% of the single Gaussian source simulation time.

In Fig. 1, the agreement between the radial photon source distributions, derived from MC calculations using our double Gaussian implementation and measurements, is very good. Small discrepancies can be observed between the tails of the source distributions. Nevertheless the agreement in this region is within 3%. The two components of the source are a relatively narrow Gaussian ($\sigma_{2}=0.077\;\text{mm}$) and a second Gaussian distribution that is much wider ($\sigma_{1}=0.334\;\text{mm}$). The latter produces an electron source distribution with a higher tail region than a single Gaussian.

The maximum difference found between measured and MC calculated OFs, using our double Gaussian source model, was of 2.5%. For the single Gaussian source model this difference increases to 9% for the smallest evaluated field. These discrepancies between measured and MC calculated OFs are more significant in the steep drop‐off zone of the curve (below the $3\times 3\;{\text{cm}}^{2}$ field), due to the partial occlusion of the radiation source, as shown in Fig. 2. The drop‐off is more pronounced for the single Gaussian source model, due to its wider source profile, compared with our double Gaussian model.

The dose profiles obtained from measurements showed a better agreement, for all field sizes, with the MC calculations using the double Gaussian source model, as compared with the single Gaussian. However, the difference between the calculated profiles decreases with increasing field size. This difference is more noticeable in the penumbra region of the profiles, whose shape is strongly dependent on the shape of the radiation source. As the photon radiation source derived from the single Gaussian model has a wider FWHM, the dose distributions obtained from this source show a wider penumbra than the dose profiles obtained from the double Gaussian source model. Additionally, it has been found that for the stereotactic collimators the differences in the dose profiles between the two MC calculations is negligible, as shown in Fig. 4. This can be understood if we consider that the penumbra extension depends also on the source‐to‐collimator distance (SCD), reducing the source occlusion influence as the SCD increases. Because the SCD for the stereotactic collimators is much greater than the SCD for the jaws and the MLC, the penumbra difference between the single and double Gaussian source models is reduced. It can be observed that the shape of the measurement is not fully reproduced by the MC modeling. However, this could be due to a partial volume effect of the detector.

The double Gaussian source model produces an underestimation of the penumbra value obtained experimentally, with mean differences of 2.3% and 2.5% in X and Y directions, respectively. Instead, for the single Gaussian source model an overestimation of the experimental penumbra is observed, with mean differences of 8.9% and 11.6% in X and Y directions, respectively. Table 1 shows that the difference between the single and double Gaussian models is more important for the jaws, because they are closer to the radiation source, and therefore more influenced by its shape, than the MLC.

This study showed that an accurate determination of the electron source helps to improve the MC modeling of the linac. The newly implemented double Gaussian source would be helpful to tune the MC model of a linac more accurately when using small fields for photon beam simulations, which would impact the results for IMRT treatments, where the beamlets used could be very narrow and thus more susceptible to the source model used. Using the new source model, this study provides useful information for understanding the dosimetric impact of the shape of the radiation source and how to implement this parameter into the BEAMnrc MC code.

## ACKNOWLEDGMENTS

The authors would like to acknowledge project FONDECYT Iniciación No. 11130575 for financial support.

## COPYRIGHT

This work is licensed under a Creative Commons Attribution 3.0 Unported License.
